# A Strange Case

**Published:** 1864-04

**Authors:** Charles E. Davis


A Strange Case. *By Charles E. Davis.*—A Physiological curiosity has just come to my knoweldge, which I think should be reported, as I do not remember to have read of a similar instance, viz., *a ruminating man.*

Mr. B. of——, R. I, presented himself for professional services yesterday, and related to me the remarkable fact that all his food, after having been swallowed, was returned to the mouth and was again masticated. This process, after a full meal, requires about four hours to complete. If I understood him aright, all matter in the stomach, not fluid or pulpy, had to be reduced to that condition before it would "stay down," any hard lumps making their presence sensibly felt in the stomach. The process of regurgitation is not under the control of the will at all, is attended by no nausea of disagreeable sensations. The food returned is sweet, and quite as palatable as when first taken from the table.

While in conversation with him, a slight motion of the chest forward, with an upward jerk of the chin, was observed, and mastication quietly commenced the work of repreparing the day's dinner for digestion. The gentleman, to convince me that he was not imposing on my credulity, took from his mouth what was raised at that time, and showed it to be bits of meat, potatoes, and turnips, being a portion of the meal made from a boiled dinner. The gentleman is about sixty years of age, of a florid complexion, has been generally healthy, and indicates it in his appearance.

If, however, his food don't return for mastication he is invariably made sick.

He cannot remember when he did not ruminate, and always had an uncontrollable habit of eating fast, was often censured for it when a boy, but could not help it.

He has a nephew and a neighbor, both living in the same town, who have this pecularity. The true ruminantiæ have four stomachs. It is probable that these individuals are constituted anatomically different from other people.

New Bedford, Mass., Feb. 5, 1864.

